# Three species of Encyrtidae (Hymenoptera, Chalcidoidea) from Mongolia, with descriptions of two new species

**DOI:** 10.3897/zookeys.1292.187191

**Published:** 2026-09-23

**Authors:** Boyu Zou, Huanji Liu, Byambasuren Mijidsuren, Yanzhou Zhang

**Affiliations:** 1 Key Laboratory of Zoological Systematics and Evolution, Institute of Zoology, Chinese Academy of Sciences, 1 West Beichen Road, Beijing 100101, China Key Laboratory of Zoological Systematics and Evolution, Institute of Zoology, Chinese Academy of Sciences Beijing China; 2 Institute of Plant Protection Research, B.O. Box-51/42, Ulaanbaatar-13330, Mongolia Institute of Plant Protection Research Ulaanbaatar Mongolia

**Keywords:** Chalcidoidea, *
Helegonatopus
*, new record, new species, parasitic wasp, taxonomy, *
Trechnites
*

## Abstract

Two species, *Trechnites bulgani* Zou & Zhang, **sp. nov**. and *Helegonatopus arkhangaiensis* Zou & Zhang, **sp. nov**. are described as new to science. *Psyllaephagus tamaricicola* Myartseva is recorded from Mongolia for the first time. A diagnosis or a description, figures of the characters, as well as the known distribution of each species, are provided.

## Introduction

Encyrtidae (Hymenoptera: Chalcidoidea) is one of the largest families of parasitic Hymenoptera (Noyes and Hayat 1984; Trjapitzin 1989), with about 4000 species included in more than 500 genera (UCD Community 2023). Encyrtidae comprises some of the most important biological control agents against phytophagous insects (Trjapitzin 1989; Noyes and Hayat 1994; Noyes and Woolley 2025). Among them, hundreds of species have been used as biological control agents of insect pests (Japoshvili and Noyes 2005).

The taxonomic study of Encyrtidae in Mongolia began in the late 1960s. Following decades of continuous investigation and taxonomic research by various authors (Hoffer 1970, 1977, 1980; Szelényi 1971, 1972, 1982; Trjapitzin 1972, 1976, 1982, 1989; Sugonjaev and Trjapitzin 1974; Myartseva 1977, 1982; Khlopunov 1980; Sakhnov 1993), approximately 100 genera and over 300 species have been recorded from the country to date (UCD Community 2023). Despite this significant progress, many regions remain insufficiently surveyed, and numerous species likely remain undiscovered. In the summer of 2025, a joint expedition conducted by the Institute of Zoology, Chinese Academy of Sciences and the Mongolia Institute of Plant Protection Research collected three species of Encyrtidae from Mongolia. After carefully comparing them with the types and descriptions of known species, we are convinced that two of the species are new to science, and their descriptions are provided in this paper. *Psyllaephagus tamaricicola* Myartseva is a new record for Mongolia.

## Materials and methods

Field surveys were conducted across Mongolia from 2023 to 2025. Systematic sampling was carried out in Mongolia, covering most of the country’s territory. Specimens were primarily collected using sweep netting and yellow pan traps.

Specimens were then card-mounted or dissected and mounted on slides, generally following the method described by Noyes (1982). Specimen observation and measurement were performed with a Nikon SMZ-168 stereomicroscope. Body lengths are given in millimetres; other measurements are relative, taken from the divisions of a linear scale of a micrometer placed in the eyepiece of a stereozoom binocular microscope at 4.7× Zoom. Microphotographs were taken with a Canon EOS550D digital camera connected to a Leica DM-2500 microscope, and photos of card-mounted specimens were produced using a Nikon D7000 digital camera coupled to a Nikon SMZ-1500 stereomicroscope. Specimens examined in this study are deposited in the following institutions: Institute of Zoology, Chinese Academy of Sciences (**IZCAS**), and the Mongolian Institute of Plant Protection Research (**MIPPR**).

Morphological terminology and abbreviations follow that of Noyes and Hanson (1996) and Gibson (1997) with some modifications. Absolute measurements were used for body length. Abbreviations used in the text are as follows: **HW**, maximum head width; **HL**, minimum head length; **FV**, the width of the frontovertex; **MS**, the malar space; **OOL**, the minimum distance between the eye margin and the nearest posterior ocellus; **POL**, the minimum distance between the posterior ocelli; **SL**, the length of the scape; **SW**, maximum width of the scape; **F1** through **F6**, 1^st^ to 6^th^ funicular segments; **MSL**, mesoscutum length; **MSW**, mesoscutum width; **MT**, mid tibia length; **MVL**, marginal vein length; **MVW**, marginal vein width; **PVL**, postmarginal vein length; **PVW**, postmarginal vein width; **SVL**, stigmal vein length; **SVW**, stigmal vein width; **OL**, ovipositor length.

## Results

### *Helegonatopus* Perkins, 1906

#### Helegonatopus arkhangaiensis

Taxon classificationAnimaliaHymenopteraEncyrtidae

Zou & Zhang sp. nov.

B29A0D16-596A-53E0-B9B4-744F6A729CF9

https://zoobank.org/2DA61674-0DBB-4173-B813-0426C72B7F63

Figs 1, 2

##### Type material.

***Holotype***. • ♀, Mongolia, Arkhangai, Tayhar, 16. VI. 2024, coll. H.J. Liu (deposited in IZCAS). ***Paratypes***. • 2♀♀, same data as holotype (1♀ deposited in IZCAS; 1♀ deposited in MIPPR).

**Figure 1.**
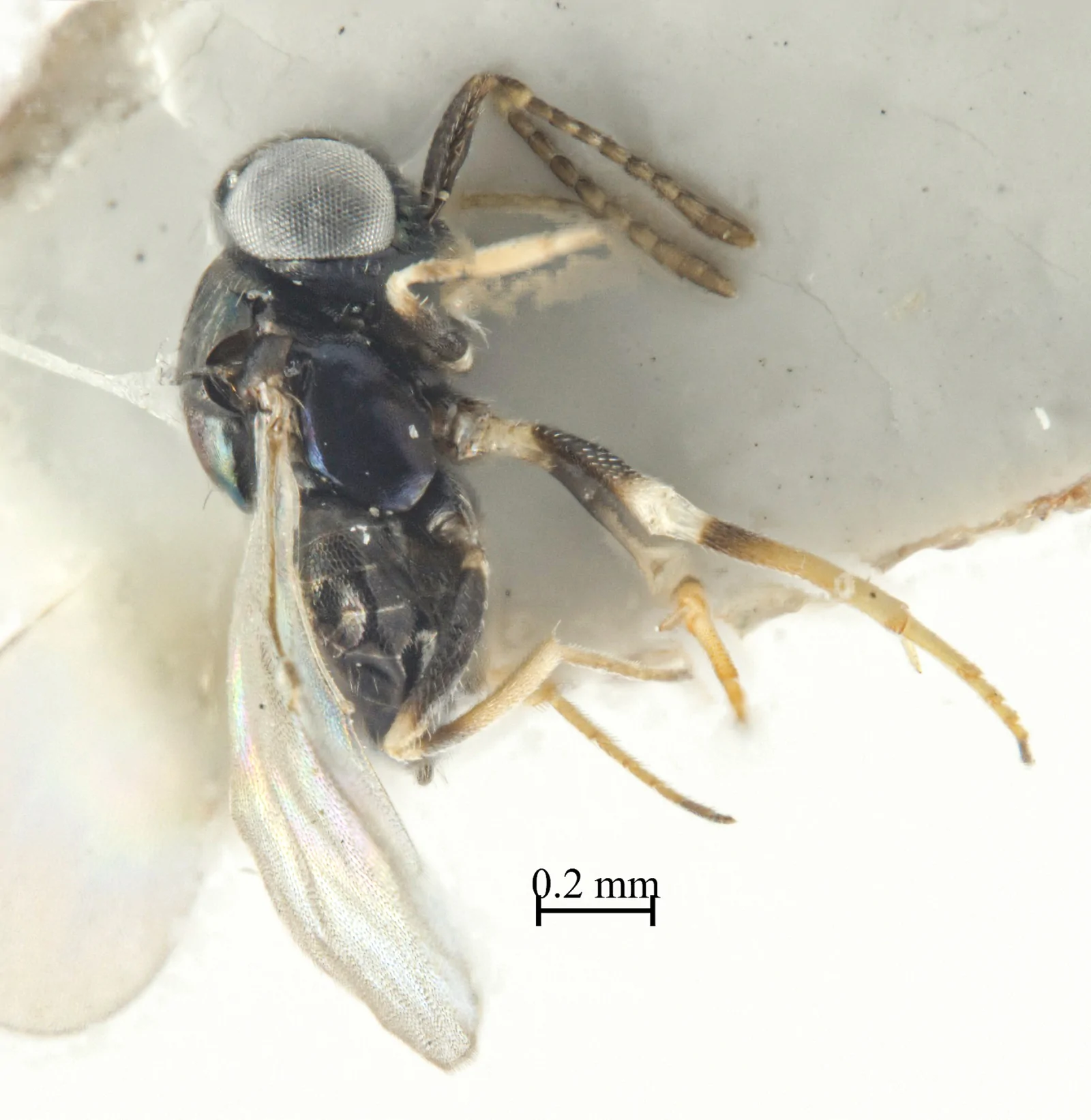
*Helegonatopus arkhangaiensis* Zou & Zhang, sp. nov. Paratype ♀. Body in lateral view.

**Figure 2.**
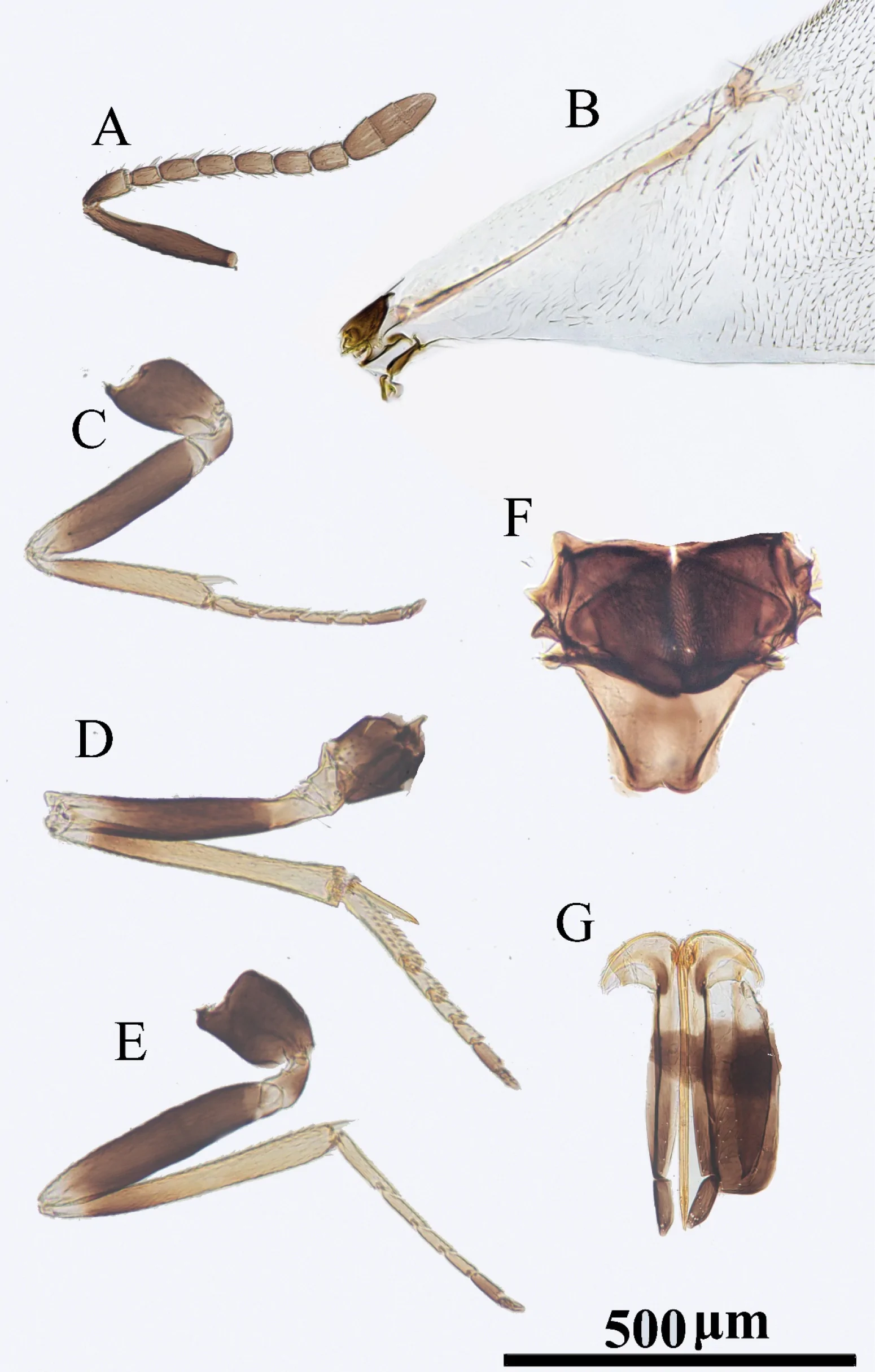
*Helegonatopus arkhangaiensis* Zou & Zhang, sp. nov., holotype, ♀. **A**. Antenna; **B**. Basal part of fore wing; **C**. Fore leg; **D**. Mid leg; **E**. Hind leg; **F**. Scutellum; **G**. Ovipositor.

##### Other material.

• 3♀♀, Mongolia, Khovd, Bulgan, 22. VII. 2025, coll. B.Y. Zou (deposited in IZCAS).

##### Diagnosis.

**Female**. Body length including ovipositor about 1.0–1.3 mm; ocelli form acute triangle, with an angle of approximately 40°; all funicle segments longer than broad; clava 3-segmented, slightly shorter than F4–F6 combined; all coxae and femora dark brown; mid and hind tibiae marked with dark brown, fore tibia with base brown; MT about as long as OL.

##### Description.

**Female** (Holotype). Length about 1.1 mm. Body generally dark brown with a faint metallic green sheen. Frontovertex and scrobe with black sheen. Mesoscutum and scutellum with dark-green sheen. Scape and pedicel dark brown, except apex brown, funicle and clava brown; tegula entirely dark brown; axilla with faint metallic dark-blue sheen; all coxae dark brown; fore and hind femur dark brown, mid femur except basal 1/5 and apex dark brown; fore and hind tibiae except apex dark brown; mid tibia with a dark brown spot at base, the rest brownish yellow; fore wings hyaline, venation brown.

***Head***. Head with reticulate sculpture on frontovertex; HW approximately 2.1× HL; FV about 0.4× HW; ocelli form obtuse triangle, with an angle of approximately 40°; OCL about 1.6× the diameter of posterior ocellus; OOL about 0.5× diameter of posterior ocellus; POL about 4× diameter of posterior ocellus; antenna with scape approximately 6.1× as long as broad; pedicel approximately 2.3× as long as broad, and slightly shorter than F1–F3 combined; all funicle segments longer than broad (F1 1.8×, F2 2.0×, F3 2.0×, F4 1.7×, F5 1.7×, F6 1.4×); clava about 2.7× as long as broad and same as long as F3–F5 combined. Relative measurements: HW 12.3, HL 5.9, FV 5.2, POL 1.8, OCL 0.7, OOL 0.2, MS 4.4, SL 10.6, SW 1.7, PL 3.0, PW 1.3.

***Mesosoma***. Mesoscutum slightly convex, covered with fine, with similar reticulate sculpture to that on frontovertex; MSW about 1.35× MSL; scutellum covered with lineolate reticulate sculpture and clearly deeper than that on mesoscutum; SVL about 6.5× SVW; axilla with very shallow reticulations; fore wing approximately 2.2× as long as broad; linea calva not closed; basal cell with a naked area at base; marginal vein punctiform, post marginal vein virtually absent; mid tibial spur about same as long as the mid basitarsus. Relative measurements: FWL 43.5, FWW 19.8, MVL 0.8, SVL 2.1.

***Metasoma***. About 1.0× as long as mesosoma; hypopygium extending to apex of gaster; ovipositor nearly as long as mid tibia, not exserted. Relative measurements: OL 20.3.

**Male**. Unknown.

##### Host.

Unknown.

##### Distribution.

Mongolia (Arkhangai, Tayhar).

##### Etymology.

The specific name refers to Arkhangai, where the type specimen was collected.

##### Comments.

*Helegonatopus arkhangaiensis* sp. nov. is morphologically similar to *H. rasnitzyni* Trjapitzin, 1963. The new species can be distinguished by the following characters: antennal clava slightly longer than F1–F3 combined (in *H. rasnitzyni*, clava about as long as F1–F5 combined); ocelli form an angle of approximately 40° (in *H. rasnitzyni*, ocelli forming an angle of slightly less than 90°).

### *Psyllaephagus* Ashmead, 1900

#### Psyllaephagus tamaricicola

Taxon classificationAnimaliaHymenopteraEncyrtidae

Myartseva, 1979

9C868136-3491-57A3-9DFB-82E8168CA82E

Fig. 3

Psyllaephagus tamaricicola Myartseva, 1979. New record for Mongolia.
Psyllaephagus tamaricicola Myartseva, 1979: 29. Holotype ♀, Turkmenistan.
Psyllaephagus tamaricicola Myartseva: Trjapitzin 1989: 256.

##### Material examined.

Mongolia • 2♀♀, Khovd, Mankhan, coll. B.Y. Zou, 17. VI. 2025 (deposited in IZCAS).

**Figure 3.**
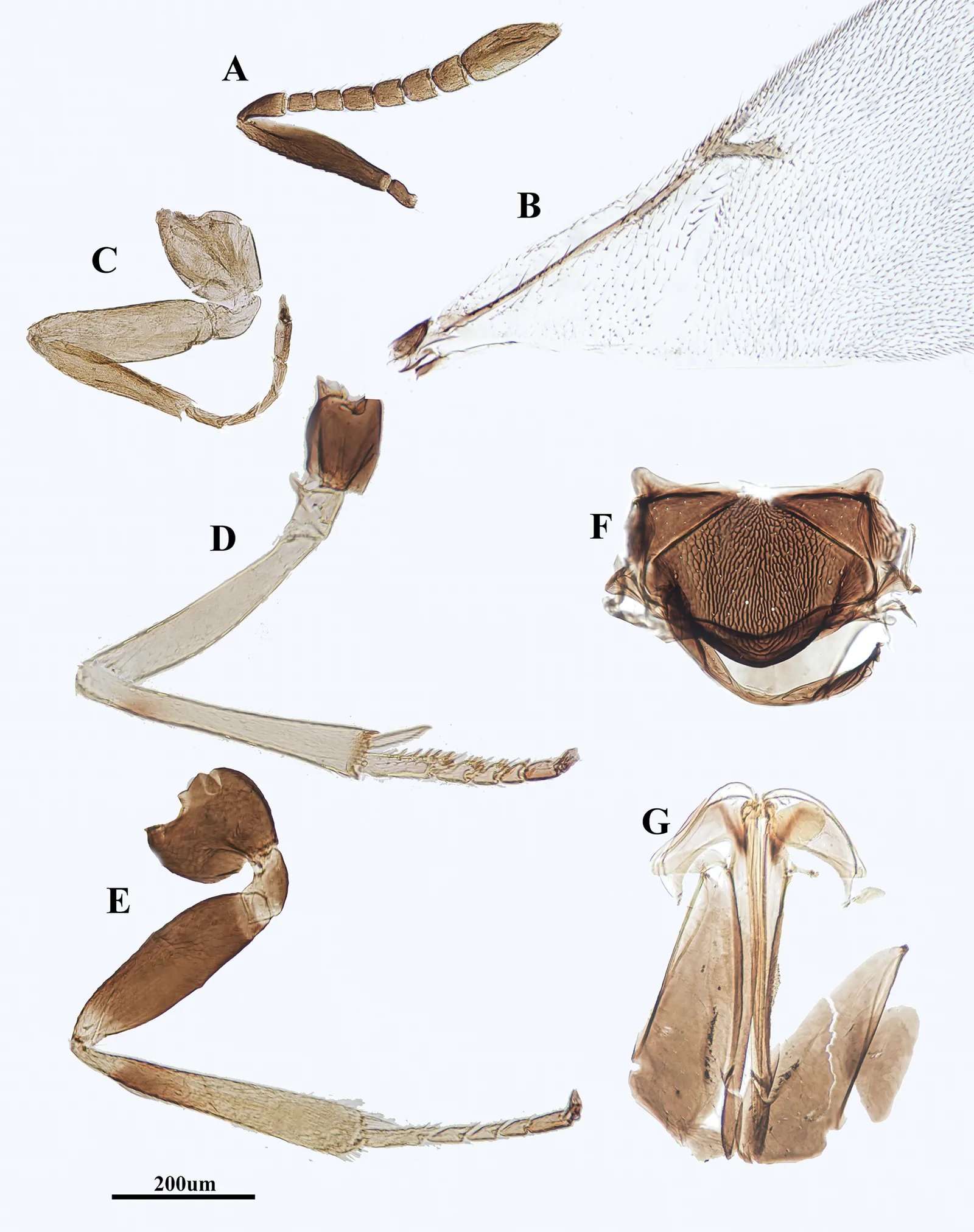
*Psyllaephagus tamaricicola* Myartseva, 1979, ♀. **A**. Antenna; **B**. Basal part of fore wing; **C**. Fore leg; **D**. Mid leg; **E**. Hind leg; **F**. Scutellum; **G**. Ovipositor.

##### Diagnosis.

**Female**. Body length including ovipositor about 1.2–1.4 mm; ocelli form an acute triangle; F1–F4 longer than broad, F5 and F6 quadrated; clava unsegmented, about as long as F4–F6 combined; fore coxae yellow, mid and hind coxae dark brown; hind femora dark brown, tibia marked with dark brown; MT about 1.3× as long as OL.

##### Description.

**Female**. Length about 1.4 mm. Body generally dark brown with a faint metallic green sheen. Frontovertex and scrobe with blue-violet sheen; mesoscutum and axillae with metallic green; scutellum with golden-green sheen; metasoma with metallic green. Scape and pedicel dark brown, funicle and clava brown; tegula pale yellow except apex dark brown; fore legs entirely yellow; mid and hind coxae dark brown; hind femora dark brown excepted apex of 1/7 yellow; hind tibia marked with dark brown at basal of 1/4; fore wings hyaline, venation brown.

***Head***. Head with reticulate sculpture on frontovertex; HW approximately 2.6× HL; FV about 0.4× HW; ocelli form obtuse triangle, with an angle of approximately 80°; OCL about 1.1× diameter of posterior ocellus; OOL about 0.5× diameter of posterior ocellus; POL about 5.3× diameter of posterior ocellus; antenna with scape approximately 4.6× as long as broad; pedicel approximately 2.0× as long as broad, and shorter than F1–F3 combined; funicle segments F1–F4 longer than broad, F5 and F6 quadrate (F1 1.5×, F2 1.5×, F3 1.4×, F4 1.2×, F5 1.0×, F6 1.0×); clava about 2.2× as long as broad and same as long as F4–F6 combined. Relative measurements: HW 27, HL 10.4, FV 9.5, POL 5.4, OCL 1.3, OOL 0.4, MS 9.5, SL 10.1, SW 2.3, PL 2.9, PW 1.6.

***Mesosoma***. Mesoscutum slightly convex, covered with fairly regular, reticulate sculpture, slightly deeper than that on frontovertex; MSW about 1.4× MSL; scutellum covered with lineolate reticulate sculpture and clearly deeper than that on mesoscutum; SVL about 2.1× SVW; axilla with thimble-like sculpture; fore wing approximately 2.5× as long as broad; linea calva closed by 1–2 lines of setae posteriorly; basal cell with a naked area at base; marginal vein punctiform, post marginal vein about 0.6× as long as stigma vein; mid tibial spur about same as long as the mid basitarsus. Relative measurements: FWL 65, FWW 39, MV 1.9, PMV 2.1, SVL 3.8.

***Metasoma***. About 1.2× as long as mesosoma; hypopygium extending to apex 2/3 of gaster; ovipositor not exserted, about 1.3× as long as mid tibia. Relative measurements: OL 25.5.

##### Host.

Unknown.

##### Distribution.

Armenia (Trjapitzin 1989); Kazakhstan (Trjapitzin 1989); Turkmenistan (Myartseva 1979); Mongolia (Kovd Mankhan).

### *Trechnites* Thomson, 1876

#### Trechnites bulgani

Taxon classificationAnimaliaHymenopteraEncyrtidae

Zou & Zhang sp. nov.

0912ABC7-4591-56C1-B0C4-68B36E405C4F

https://zoobank.org/C0C9BBDD-3950-406D-BD5D-61330584E0DC

Figs 4, 5

##### Type material.

***Holotype***. • ♀, Mongolia, Khovd, Bulgan, 22. VII. 2025, coll. B.Y. Zou (deposited in IZCAS). ***Paratypes***. • 4♀♀, same data as holotype (2♀♀ deposited in IZCAS; 2♀♀ deposited in MIPPR).

**Figure 4.**
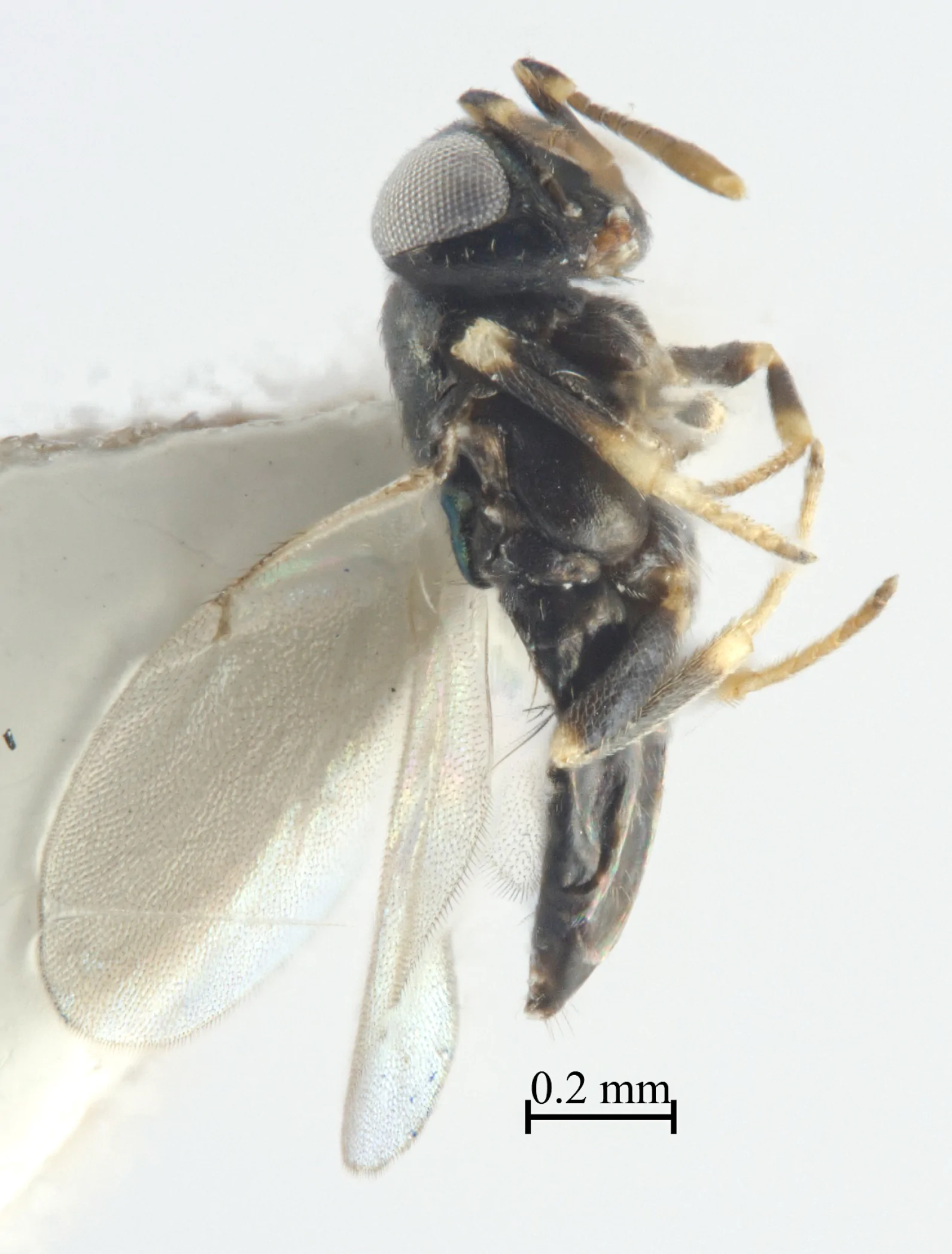
*Trechnites bulgani* Zou & Zhang, sp. nov. Paratype ♀. Body in lateral view.

**Figure 5.**
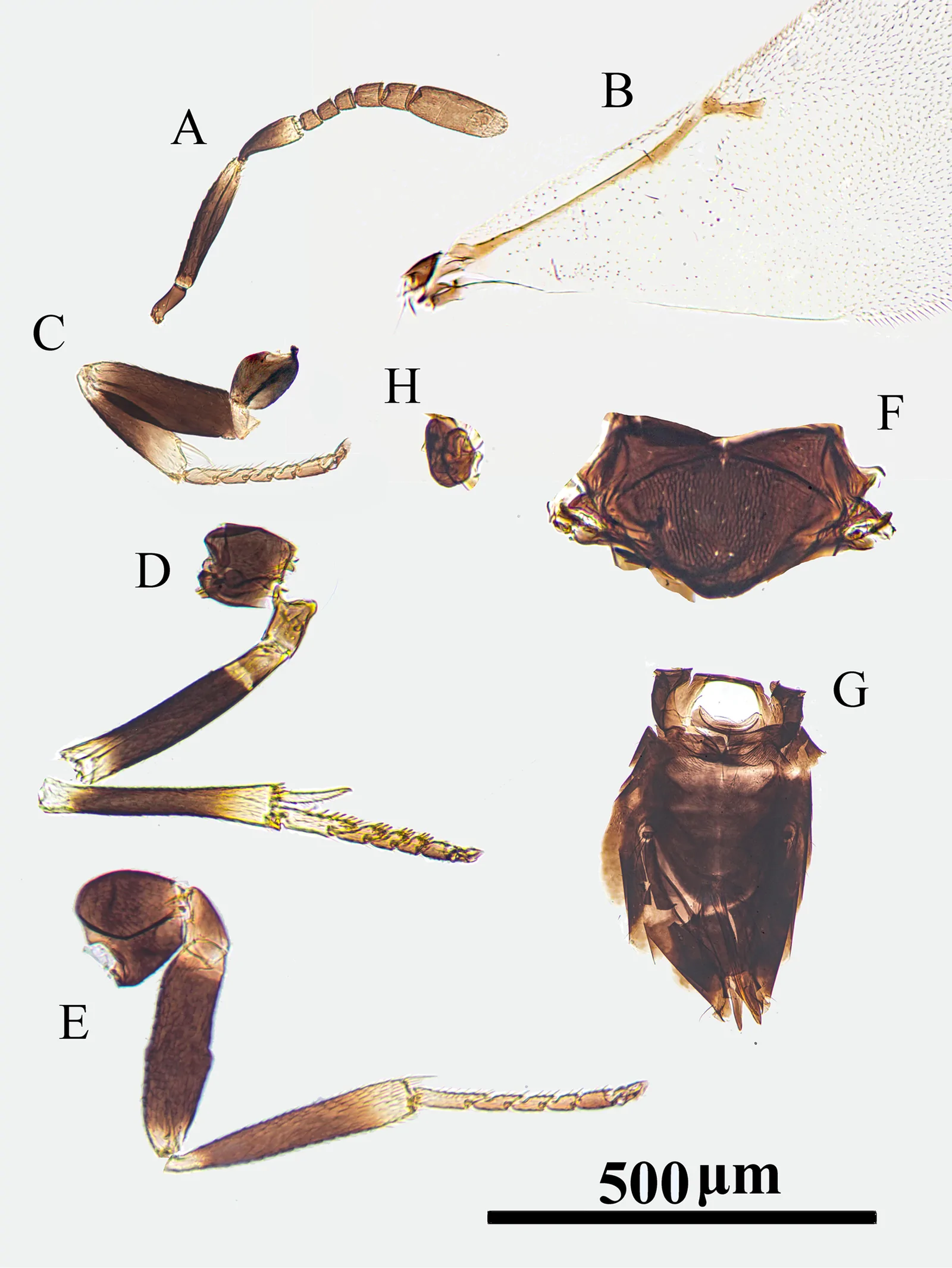
*Trechnites bulgani* Zou & Zhang, sp. nov., holotype, ♀. **A**. Antenna; **B**. Basal part of fore wing; **C**. Fore leg; **D**. Mid leg; **E**. Hind leg; **F**. Scutellum; **G**. Gaster; **H**. Tegula.

##### Diagnosis.

**Female**. Body length including ovipositor about 1.0–1.1 mm; ocelli form obtuse triangle, with an angle of approximately 110°; funicle segments only F4 longer than broad, the rest segments subquadrate; clava 3-segmented, about same as long as F2–F5 combined; notauli absent; all coxae and femora dark brown; mid tibia marked with dark brown, fore and hind tibiae dark brown; MT about 1.6× as long as OL.

##### Description.

**Female** (Holotype). Length about 1.1 mm. Body generally dark brown with a faint metallic dark sheen on mesoscutum; scutellum with metallic blue reflections contrasting with the dark mesoscutum. Scape and pedicel dark brown, except apex brown, funicle and clava brown; tegula entirely dark brown; axilla with faint metallic dark-blue sheen; all coxae dark brown; all femora except apex, dark brown; fore, mid and hind tibiae except apex dark brown; fore wings hyaline, venation brown.

***Head***. Head covered with reticulate sculpture; HW approximately 2.8× HL; FV about 0.3× HW; ocelli form obtuse triangle, with an angle of approximately 110°; OCL about 1.0× diameter of posterior ocellus; OOL about 0.45× diameter of posterior ocellus; POL about 5.1× diameter of posterior ocellus; antenna with scape approximately 4.9× as long as broad; pedicel approximately 2.5× as long as broad, and slightly longer than F1–F3 combined; funicle segments F3, F4 and F5 slightly longer than broad, F1 quadrate, F2 slightly broader than long (F1 1.0×, F2 0.88×, F3 1.1×, F4 1.33×, F5 1.1×); clava about 2.7× as long as broad and about same as long as F2–F5 combined. Relative measurements: HW 17.3, HL 9.5, FV 5.2, POL 3.1, OCL 0.6, OOL 0.3, MS 4, SL 7.1, SW 1.1, PL 4.7, PW 2.0.

***Mesosoma***. Mesoscutum slightly convex, covered with fine, reticulate sculpture; notaular lines absent; MSW about 1.5× MSL; scutellum covered with lineolate reticulate sculpture and clearly deeper than that on mesoscutum; SVL about 2.5× SVW; axilla with very shallow reticulations; fore wing approximately 2.5× as long as broad; linea calva closed by 1–2 lines of setae posteriorly; basal cell with a naked area at base; marginal vein punctiform, post marginal vein virtually absent; mid tibia spurs about 0.8× of mid basitarsus. Relative measurements: FWL 49, FWW 20, MVL 1.1, SVL 2.2.

***Metasoma***. About 1.2× as long as mesosoma; hypopygium reaching to 3/4 of gaster; ovipositor nearly as long as mid tibia, slightly exserted. Relative measurements: OL 7.3.

**Male**. Unknown.

##### Host.

Unknown.

##### Distribution.

Mongolia (Kovd, Bulgan).

##### Etymology.

The specific name refers to Bulgan, where the type specimen was collected.

##### Comments.

*Trechnites bulgani* sp. nov. is morphologically very similar to *Trechnites alni*Erdös 1957, but can be distinguished by the following characters: scape and pedicel with pale apices (in *T. alni*, antennal scape and pedicel completely dark); the new species with notauli absent (in *T. alni*, notauli reach about 1/3 across mesoscutum).

## Supplementary Material

XML Treatment for Helegonatopus arkhangaiensis

XML Treatment for Psyllaephagus tamaricicola

XML Treatment for Trechnites bulgani

## Additional information

### Artificial Intelligence (AI) use

The authors accept full responsibility for the content of the manuscript, including the disclosure of any use of AI.

No AI tools were used in the preparation of this manuscript.

### Author ORCIDs

B. Zou https://orcid.org/0009-0000-5022-8669

H. Liu https://orcid.org/0009-0000-3128-5684

B. Mijidsuren https://orcid.org/0000-0002-7806-2039

Y. Zhang https://orcid.org/0000-0002-9713-5352

### Data availability

All of the data that support the findings of this study are available in the main text.
